# The White Thrombus a Novel Primary Affection

**Published:** 1889-03

**Authors:** 


THE WHITE THROMBUS A NOVEL PRIMARY AFFECTION.—In a lecture before the Verein fuer innere Medicin, in Berlin, Prof. Litten related, December 3, 1888, the case of a young man who died with an uncontrollable bloody diarrhea in the short space of three days. The intestinal canal was in a state of bloody infiltration, the cause of which was found in a thrombus of perfectly white appearance, ten cm. from the aorta in the arteria mesaraica superior. Peripherally of this thrombus was a red one. The white thrombus was evidently primarily white, altogether formed from the third blood element (Bizzozeros plates) in a state of disintegration. The endothelium of the artery was lost, and the thrombus quite solidly attached. Of course, the location caused the issue. Seemingly the loss of the endothelium was primary, but why? No atheromatous changes elsewhere; valves of the heart normal.—*Vide Berlin. klin. Wochenschr.*, No. 1, page 15. 1889.

